# Dressing disrupted: negotiating care through the materiality of dress in the context of dementia

**DOI:** 10.1111/1467-9566.12575

**Published:** 2018-02-21

**Authors:** Christina Buse, Julia Twigg

**Affiliations:** ^1^ Department of Sociology University of York UK; ^2^ Social Policy, Sociology and Social Research University of Kent Canterbury UK

**Keywords:** dementia, clothing, embodiment, bodywork, material culture

## Abstract

This paper explores how the materiality of dress mediates and shapes practices of care in the context of dementia. Earlier research called for an approach to conceptualising care that recognised the role played by everyday artefacts. We extend this to a consideration of dress and dressing the body in relation to people with dementia that involves the direct manipulation of material objects, as well as the materiality of bodies. The paper draws on an ESRC funded study Dementia and Dress, which examined experiences of dress for people with dementia, families and care‐workers using ethnographic and qualitative methods. Our analysis explores the process of dressing the body, the physicality of guiding and manipulating bodies into clothing, dealing with fabrics and bodies which ‘act back’ and are resistant to the process of dressing. We consider how the materiality of clothing can constrain or enable practices of care, exploring tensions between garments that support ease of dressing and those that sustain identity. Examining negotiations around dress also reveals tensions between competing ‘logics’ of care (Mol 2008).

## Introduction

Sociological research has increasingly addressed the active role played by everyday artefacts in mediating practices of care (Mol 2008, Mol *et al*. 2010, Pink *et al*. 2014, Schillmeier and Domènech 2009). The role of dress, however, has received limited attention within the sociology of health and illness. Dress studies in turn have also neglected the field of care, preferring to focus on the fashionable, the agentic and subversive. And yet dress represents a significant part of the care environment, with potential to shape experiences of care and possibilities for identity construction. In this article we aim to create dialogue between these two areas of literature, arguing that attending to dress practice can shed light on practices and experiences of care.

The article draws on an innovative ESRC funded study, Dementia and Dress, which sought to examine questions of identity, personhood, embodiment and relationships in dementia through a material analysis of dress. The study aimed to assess the significance of dress in the day‐to‐day lives of people with dementia, and those who support them. It aimed to go beyond a simple focus on service delivery, to explore everyday embodied experiences of care, drawing on the wider literature of embodiment in order to shed new light on experiences of dementia (Kontos 2004, Martin and Kontos 2013). Elsewhere we have focused on the perspective of the person with dementia (Buse and Twigg 2015, 2016). Here we focus on the accounts of care‐workers, and the active role of dress in care practice.

We begin with a brief discussion bringing together literatures on care, material culture and dress. We then present a detailed analysis of the act of dressing in the context of care, drawing on data from the Dementia and Dress study. This is followed by exploration of how choices are made, and the significance of clothing for identity in dementia care settings. We consider how the materiality of clothing can constrain or enable practices of care, exploring tensions between garments that support ease of dressing and those that sustain identity. We then consider the role of dress in the aesthetics of care, as a visible indicator of care quality (Ward *et al*. 2008), and how it is entangled with different ‘logics of care’ (Mol 2008). We suggest that dress as an aspect of material culture shapes how care is ‘felt and lived’ in everyday contexts (Lavis *et al*. 2015: 4).

## Care, clothing and material culture

Care is a multi‐faceted concept, it has been theorised as an emotion or a feeling, a relationship, a form of labour, action or performance (Lavis *et al*. 2015, Rummary and Fine 2012). In this article we focus on care as an embodied practice, drawing on the concept of ‘bodywork.’ Bodywork is defined by Twigg *et al*. (2011: 171) as paid work that ‘focuses directly on the bodies of others: assessing, diagnosing, handling, treating, manipulating, and monitoring bodies.’ The concept is useful for understanding the labour of care‐workers, highlighting the embodied aspects of their work. Care work is low status and low paid work, that is also transgressive and ‘dirty’, involving dealing with unbounded, leaky bodies (Lawton 1998).

The bodywork of care is also closely linked to emotion and to emotional labour (England and Dyck 2011, Lee‐Treweek 1996). Emotional labour is defined as the ‘management of feeling to create a publicly observable facial and bodily display’ (Hochschild 1983/2012: 29), in accordance with occupational ‘feeling rules’. The intimacy of care transactions, their close tactility and capacity for transgression, mean that emotions are indeed often powerfully evoked, deployed and supressed as part of the work. Research on bodywork has also drawn attention to material things, for instance, the use of gloves as a strategy for managing the transgressive aspects of care work, and providing physical and symbolic distance from the body (Twigg 2000).

Research on material culture and healthcare has further illuminated the tactile and multi‐sensory elements of care practices. For instance, Pink *et al*. (2014: 427) examine how mundane objects like gloves, soap and hand gel are embedded within health care‐workers practices of ‘tactile knowing’. Latimer (2003) employed an ethnographic analysis of everyday things – sluice pans, towels, washing bowls, cotton balls – to explore how nurses perform their identity through the materials they work with. At the same time, research drawing on science and technology studies has explored the entanglement of people and things in practices of care (Mol *et al*. 2010, Schillmeier and Domènech 2009), defining care as relational and processual (Latimer 2013).

Though literature on the bodywork of care has not focused specifically on dress, the subject emerges in accounts of care routines. For example, Lee‐Treweek's (1997) classic analysis of the production of the ‘lounge‐standard’ resident, centres around imposing normative ideas of femininity on the bodies of residents. At the same time, historical research on institutional dress has highlighted the role of standardised dress in Othering, and disciplining the body in institutional contexts (Linthicum 2006). Finnish research on hospital dress has drawn attention to its potential role in the reproduction of patienthood (Topo and Iltanen‐Tahkavuori 2010).

One aspect of mundane dress practice is the act of getting dressed. Entwistle (2000) drawing on Mauss (1979), describes the act of ‘getting dressed’ as a body technique ‘preparing the body for the social world’ (p.11). Banim *et al*. (2001) and Woodward (2007) similarly focus on the everyday act of choosing clothes, conceptualising it as the ‘wardrobe moment’. They consider dressing as a practice of identity construction, as the wearer considers how they want to appear to others. Woodward (2007) draws particular attention to the material properties of dress, the way clothing ‘feels’ on the body, and the significance of colours, textures, cut and style as part of the wearer's ‘personal aesthetic’, suggesting that ‘clothing materializes questions of identity in a particularly intimate way’ (p.3).

In the context of illness, disability, and care, these mundane practices of dressing take on new meaning and significance. Hayman (2009) draws attention to the practical, embodied difficulties associated with dressing in the context of disability, as well as the symbolic challenges presented by assistance that disrupts dressing as a private and autonomous act. Van Wersch (2001) explored how taken‐for‐granted practices suddenly became problematic and painful following a mastectomy, so that a desire to maintain continuity of identity through dress often underpinned decisions about using prosthetics or undergoing reconstructive surgery. Candy (2007) in her work on women with rheumatoid arthritis suggests how ‘little research has considered the contents of wardrobes as manifestations of health and illness’ (p. 3).

We seek to extend these debates through a detailed examination of the act of dressing in the context of dementia and institutional care. Dementia disrupts our taken‐for‐granted relationship to everyday objects, including clothing (Phinney and Chesla 2003, Schillmeier 2014). Common problems include: difficulties with sequencing and the order of dressing; forgetting to change clothes; and difficulties with fastenings (Feyereisen *et al*. 1999). Dementia introduces additional complexity when the person is no longer able to articulate their decisions about dress. The progression of dementia into the more advanced stages is commonly associated with a loss of interest in appearance and dress, raising questions about whether attention to appearance is for the benefit of the person with dementia, or their family. Through examining the ways in which dementia troubles dressing practice, we seek to shed light on wider debates and meanings of care.

## Methodology

The article draws on an ESRC funded study Dementia and Dress which explored the significance of clothing for people with dementia, their carers and care‐workers. Ethical approval was granted by the Social Care Research Ethics committee (SCREC). Every effort was made to involve the person with dementia in the consent process, explaining the study verbally as well as using adapted information sheets with visual images. According to the Mental Capacity Act (Department of Health 2005), capacity is defined as the person's ability to understand information about the research, retain information to consider if they wish to take part, weigh up the consequences, and communicate their decision. This was ascertained through interaction with the person with dementia, and consultation with care home managers and relatives (Hubbard *et al*. 2003). For people who lacked capacity, we identified a personal consultee, generally a family member. Consent was treated as ongoing, with the researcher continuously assessing the willingness of the person to be involved.

The research was conducted across three Kent care homes, and fifteen domestic households. The sample included thirty‐two case studies of people with dementia: fifteen living in their own homes, and seventeen in the care home settings. The three care homes were selected to reflect variation in ownership and practice, and included private and voluntary sector run homes. People with dementia were sampled purposively, to explore differences relating to class, gender, and stage of dementia, and included nine men and twenty‐three women, from different occupational backgrounds, and at different stages of dementia (from mild to severe).

The study also included interviews and observations with twenty‐nine family carers and relatives, and twenty‐eight members of care home staff (care‐workers, managers and laundry workers). This paper focuses on the responses of workers and managers in the homes. Workers ranged from age 28‐66, and their time in the care sector ranged from 56 years to 6 months. Their time working in the particular care home we visited varied from 6 months to 16 years – something which had important implications for relationships with residents and attention to dress, a point we return to. All had relevant vocational qualifications. Twenty care‐workers were white British, three were British Asian, and others had emigrated from countries including France, Poland, the Philippines, and Mauritius.

The study used ethnographic methods, including observations, qualitative interviews and ‘wardrobe interviews’ (Banim and Guy 2001: 218). Observations facilitated inclusion of the perspectives of people with more advanced dementia, who were unable to participate in formal interviews (Hubbard *et al*. 2003). In this paper, we focus on interactions with care home staff, including observations, informal ‘ad hoc’ discussions, and qualitative interviews. Qualitative interviews with staff were conducted on site at the care homes, where possible in an empty room such as the activities room, manager's office or staff room, but sometimes in a quiet corner of a communal space, due to the difficulties for staff of ‘coming off the floor’. Due to SCREC requirements, dressing the body could not be observed in the private space of the bedroom, although assistance with dress, interactions, and discussions around dress were observed within communal areas of the care homes.

The data were analysed using qualitative thematic analysis. Initial analysis took place following each fieldwork session, as part of typing up detailed field‐notes. Formal analysis began with carefully reading and re‐reading transcripts, noting down emergent concepts and categories. These initial codes were then interrogated by the researchers in light of central questions around personhood, identity, and embodiment, and similar codes were grouped into larger thematic categories. This list of themes was then used to code transcripts and fieldnotes, assisted by NVIVO qualitative software. In keeping with qualitative and ethnographic methods, analysis was iterative and ongoing.

## Dressing the body in dementia care

Dressing the body involves a ‘multiplicity of decisions, choices and physical actions’, working with ‘two materialities of very different substance and in constant juxtaposition, body and cloth’ (Hayman 2009: 628). As with other forms of practical reason and ‘body techniques’ (Mauss 1979), dressing becomes automatic and pre‐reflexive. However, dementia can disrupt this, with the result that care‐workers become increasingly involved in practices of dressing. In the earlier stages, this means ‘reminding’ and ‘prompting’ the person, helping them to pick out clothes, being on hand to assist with ‘difficult’ garments. As the condition progresses, assistance becomes more directly physical, involving the manipulation of bodies and garments:

… if you imagine you're getting someone dressed and you can say to them, “Lean forward. Can you put your arm through there and arm through there and just pull it down?” it's a lot quicker than saying to someone, “I've got to put your top on, can you lean forward?” You know; “Ethel, I need to bring your arm through, can you relax your arm, love? Can I bring it through?” and then it's very slow … and you have to be reassuring and calm. [Lisa, care‐worker]

As this care‐worker describes, dressing the body of someone with advanced dementia involves a careful process of physically manoeuvring the body into clothes, whilst providing verbal reassurance. The physicality of this process can be challenging. The bodies of people with dementia can react in unpredictable ways (Martin and Kontos 2013), ‘stiffening up’, or not ‘moving’ with the process of dressing. Some were unable to move due to physical ailments, whereas others were described as being ‘unsure of what to do with their bodies’, ‘forgetting how’ to use them.

The process of dressing was also shaped and constrained by the materiality of dress. Different garments necessitate different bodily movements, some of which are more difficult than others. Footwear, socks and tights were often described by care‐workers as ‘tricky’, both in terms of getting the person with dementia to ‘push their foot in’, and physically ‘getting [the tights or socks] up their legs’ or onto their feet, ‘especially if they're not moving themselves and you have to stand them up.’ Jumpers or tops without fastenings mean ‘putting someone's head through’ which was difficult, and could be distressing for people with dementia, producing a sensation of feeling trapped in the garment, unable to see or find their way out. Tight clothes and stiff fabrics with no ‘give’ were particularly difficult, as you could not ‘stretch them out to put them on’, and they gave no ‘room’ for the care‐worker to ‘manoeuvre’ clothing onto unbending limbs.

Dealing with these difficult aspects of dress can necessitate reducing the body to a discrete set of parts (Twigg *et al*. 2011: 172): putting someone's ‘head through’ a jumper, bringing their ‘arm through’, encouraging them to ‘push their foot in’. The descriptions above evoke images of dressing a shop mannequin, an objectified body, which is stiff and unyielding, and is dressed by removing then reattaching individual body parts. In the context of the time pressures in care homes, this reinforces the task‐orientated nature of dressing. Despite a rhetoric of choice and personalised routines, care‐workers described the pressure to have residents ‘ready for breakfast at nine o'clock’, dressed and groomed to ‘lounge standard’ (Lee‐Treweek 1997). In morning routines, activities concerned with maintaining physical health would sometimes take priority over dress, as care home manager Anita describes:

… it's about … what is a carer's priority in the day […] making sure people have eaten, washed, breathing, you know, it's the physical survival of people, isn't it, in reality!

Such competing time pressures are at odds with the natural temporality of dressing the body of someone with advanced dementia, which requires a slow and careful process.

The focus on distinct and sometimes problematic parts of the body had to be interwoven with a response based on emotional rapport, talking the person through the process of dressing in a calm and reassuring way. As one care‐worker, Kim said ‘you need to be communicating that to them at all times’ because ‘you are alleviating their fear and their anxiety all the time when you are continuously communicating.’ This suggests a ‘complex interweaving’ of ‘the physical and affective dimensions’ of care (England and Dyck, 2011: 212), in contrast to the ‘depersonalisation’ and detachment described by Lee‐Treweek (1997). The act of dressing thus requires care‐workers to move between different ontologies of bodies (Mol 2002), caught ‘between processing the body as an object and interacting with it as a materialization of personhood’ (Wolkowitz 2002: 505).

The need for emotion work – persuading, coaxing, encouraging – reflects the transgressive nature of assistance with dress. Dressing ‘involves multiple layers of privacy’ (Hayman 2009: 635) reflecting both ‘body privacy’ and ‘place privacy’ (Wiersma and Dupuis 2010: 282). Some people with dementia or their families talked about how they had grown to ‘accept’ assistance with changing clothes, despite their initial reluctance. However, assistance with dress was felt to be particularly intrusive in the more advanced stages of dementia, where it was harder to explain the process:

It's just that privacy isn't it? They think we're sort of pulling their clothes down … it's the same like you, isn't it, really? Somebody's trying to take your clothes off and you don't understand it, you will go like that [stiffen and close in limbs], won't you?”  [Mandy, care‐worker]

Removing clothing was described as particularly ‘distressing’, violating norms of bodily privacy, and disrupting the security engendered by the feel of clothing enveloping the body. This removal was sometimes resisted by the person with dementia, ‘scrunching up’ ‘stiffening’ or ‘closing in’ their limbs. Refusing to change clothes in particular necessitated careful intervention, calming and soothing residents, explaining to them what was happening: as care‐worker Mandy said ‘we just talk to them as we go along then it does come nice’, or we ‘leave them for a little while’ until ‘their moods change’. Care‐workers sometimes called in ‘someone different’ whom the person trusted. There could be a gendered dimension to this: some female residents preferred to have a female care‐worker, reflecting gendered expectations around care, and access to the female body. There could also be an element of manipulation with workers using emotion as a means of ‘getting the job done’ (Lee‐Treweek 1996). However, we also observed genuine relationships of mutual affection.

Dress could facilitate positive social interactions, with some care‐workers describing dressing as a time for ‘chat’ and ‘one‐to‐one’ interaction. This was valued by residents. Rita talked fondly about of her care‐worker Darren describing him as a ‘real gentleman.’ Some care‐workers made a point of complimenting residents on how they looked, and said that residents with dementia ‐ particularly women ‐ would ‘light up’ or ‘grow an inch’ on receiving a compliment. We also observed care‐workers using clothes as a ‘talking point’ and a way of relating to residents with more advanced dementia at a sensory level, who would ‘respond to different textures’ and ‘the feel’ and ‘touch’ of tactile fabrics like velvet and silk. In this way the material properties of dress can afford possibilities for relating to someone with dementia as a person, rather than a body to be processed (Kelly 2014).

## Choosing clothes, negotiating identities

We now explore how clothing choices are made during the process of dressing, and the potential of this for supporting or undermining identity. As we have noted, dress and personal identity are intimately connected (Banim and Guy 2001, Woodward 2007). Clothing choices are, however, always relational, shaped by social expectations, and the imagined gaze of others. This is made more complicated in the context of dementia, where care‐workers and families are drawn into the decision making, leading to a complex blurring of identities and aesthetics.

Most care‐workers emphasised the importance of enabling residents with dementia to choose their own clothing where possible. This reflects the policies of individual care homes, expressed by care home managers, and endorses the wider policy rhetoric of choice and personalisation (Mol 2008). One resident, Rita, talked about how care‐workers would involve her in decision making: ‘they take it off the coat hanger there … put coat hanger out and say, “Would you like to wear this today?”’ As dementia became more advanced, involving the person in decision making became more challenging, although it was often argued that even residents with quite advanced dementia could still be offered a ‘structured’ or ‘simplified’ choice. This involved holding up two outfits, or holding out an item of clothing, and monitoring non‐verbal responses:

Sometimes, I suppose if you hold something up and say, “Do you like this?” she might reach out and … smile or something so you could take that as a yes … We tend to do that because its conversation, you know, sort of it's nice … involving her in it. [Lisa, care‐worker]

While the person's preference might not always be clear, involving them in the process of dressing affirms identity and personhood. But such practices could be undermined by time‐pressures. Though care‐workers supported the expression of choice, as the condition progressed, they often found themselves drawn into making decisions themselves. Some felt that the focus at this stage should be on maintaining continuity with earlier identity:

I remember from when I first started what she was like. She was like a real sort of lady … so I sort of keep that in mind and get things that I know … she … as she was back then would like to be in … [Darren, care‐worker]

In this way care‐workers often became involved in the process of ‘curating’ identity on behalf of the person with dementia (Crichton and Koch 2007). This involved paying attention to maintaining the ‘little’ aspects of dress, and trying to maintain consistency with that person's embodied biography and personal aesthetic, rather than imposing a homogeneous appearance. For Darren, these practices were embedded within a long period of knowing and working with residents. Such careful attention to dress was generally more common among longer established care‐workers, pointing to the problems that high staff turn‐over rates and increased use of agency workers present for quality of care. Nonetheless, care‐workers with a less longstanding relationship with residents could draw on written information in care plans, or stories from family carers. Garments also acted as material clues, particularly where the person had no relatives: as one care‐worker said ‘you see what they've got when they come in’.

Identity thus becomes relationally constructed between the materiality of dress and the bodies of care‐workers, families, and people with dementia, a process of ‘becoming’ or ‘being‐with’ (Latimer 2013: 37). Care‐workers often described how in deciding what the person should wear, they engaged in ‘empathic reflection’ (Kelly 2014: 5), thinking about how clothes were important to them, and how they might feel ‘in their shoes’. Such reflection promotes consideration of ‘commonalities of selfhood’, supporting person‐centred approaches to care (Kelly 2014). However, it could sometimes lead to presumptions about what was aesthetically preferable. As a care home manager Anita said: ‘you do have to remind staff, you know: Dress the person as they want to be dressed and choose to dress rather than what you think looked nice’.

The construction of identity through dress is not only about appearance, but what ‘feels right’ on the body, and notions of comfort, as a ‘physical sensation’ and ‘aesthetic fit’ – ‘the wearing of clothes which are ‘you’’ (Woodward 2007: 73). People with dementia described complex and varied notions of ‘comfort’, which reflected classed and gendered identities, as well as their ‘personal aesthetic’ (see Buse and Twigg 2015). For instance, one woman said ‘I never wore trousers […] I could never feel comfortable going out in trousers’; while one man said he had ‘just felt right’ in a shirt and tie. However, care‐workers often defined comfort in terms of loose fitting clothing, elasticated waists, and casual dress, which accommodated changing bodies and care needs. Stretchy fabrics made the experience of dressing and undressing easier: ‘you know, sort of material that gives because it's easier and it's more comfortable for them’. Comfort was also interpreted in terms of casual dress, like track‐suit bottoms:

… they don't want all these tight trousers round their bellies and that sort of thing. A couple of tracksuit bottoms and their little shirt or jumper, they're quite happy with what they're wearing … for comfort for them sitting about all day long. … I mean you get a trouser sort of tight up here, it's not very comfortable for them, is it?  [Helen, care‐worker]

These ideas of comfort relate to fabrics and clothing styles which accommodate more ‘static’ bodies. The homes sometimes encouraged families to buy items such as tracksuit bottoms which the person might not have worn in the past, because they were ‘comfortable’ for ‘sitting about’, as well as facilitating the process of dressing.

The capacity to maintain identity through dress was also constrained by the realities of what was in people's wardrobes. This was the product of a complex of influences: financial, relational and practical. In this the capacity of the laundry system in the home was one of the more intractable. Managers often discouraged people from bringing in woollens or delicate fabrics because of ‘bulk’ washing, and infection control requirements for high temperatures that could ‘ruin’ clothing. Care home manager Janice described this as ‘a real shame’ for ‘individuals that maybe have liked woollens to be proper woollens…proper fabrics, and silk and things like that.’ Laundry regimes and standardised regulatory requirements could therefore undermine comfort in terms of ‘clothing's affecting relationship with the body’ (Candy 2005: 2), as a sensory atmosphere, and the ‘environment closest in’ (Twigg 2010: 226).

## Dress and the aesthetics of care

Dress choices were not only entangled with constructions of identity, but sometimes came to represent a visual indicator of care quality (Ward *et al*. 2008), from the perspective of both relatives and bodies such as the Care Quality Commission (CQC). As care manager Anita said: ‘it's a real indicator for a manager to show that your residents are well cared for if they're looking clean and tidy and in clean clothes, clothes that fit them.’ The dress of residents was therefore not only subject to surveillance from visitors, but also judgements from senior staff. Care‐workers thus described dress as a ‘reflection on you’.

Care‐workers also judged one another, with the dress of residents being read as evidence of good or bad care in practice (Schillmeier 2014). Staff described other workers who dressed residents carelessly, or in ways which were at odds with their embodied identity: ‘you get some carers that'll just go to the wardrobe, grab the first thing out and shove it on them’. Small things like the *way* clothing was arranged on the body could be significant. For instance, one care‐worker dressed several women with their blouses tucked tightly into their trousers, creating an unflattering silhouette, which emphasised spreading waistlines, and a lack of supportive underwear. A worker remarked to the researcher that this looked ‘terrible’, and that the appearance of residents really ‘depends on what carer they've got.’

Poorly dressed residents could also be seen as a reflection on the habitus and ‘tastes’ of care‐workers, with a lack of fit between the habitus of staff and residents, for instance, along generational lines: ‘young carers, won't think to put a vest on an old gentleman’. Judgements about ‘taste’ were often racialized, with some care‐workers commenting on ‘foreign’ staff with a different ‘cultural’ background whose approach to dressing was not in keeping with that of (white) residents.

The quality of care was not only judged through the visual appearance of dress, but also its material and sensory properties, with care‐workers emphasising the importance of clothing which looked and smelled ‘clean’, as well as being ‘co‐ordinated’ and ‘tidy’. This could be disrupted by unruly clothing which was stained, torn or tatty:

a pair of tights … that have got a rip … a ladder in them or something … Or a skirt that's got a hole in or a jumper so, you know, I think it's not good for them to have tatty clothing on because it looks like we don't care, I think. [Ruth, registered geriatric nurse]

This avoidance of tatty, untidy clothing was partly about protecting the person with dementia from images of dereliction associated with frail old age (Twigg 2013). However, it is also about poor dress as a reflection on the care‐workers, which ‘looks like we don't care.’

Dress as an indicator of care quality, however, could clash with rhetorics of personalisation, with ‘choice’ presenting dilemmas for care‐workers:

…. it can be quite difficult because the theory is it is their choice … like Valerie; you know, some days she'll go around in those slipper things and the dressing gown. I don't know whether it's just that she can't be bothered … I mean it's obviously comfy. But then of course if the family come and *well mother's not dressed,* you know, it looks like we haven't bothered … [Carol, care home worker]

Here the care home worker is torn between conflicting logics of ‘care’ and ‘choice’ (Mol 2008), and between an emphasis on the aesthetics of care (Pols 2013) and notions of care as ‘comfort’. As discussed above, care‐workers often emphasised the importance of providing ‘comfort’ through the experience and ‘feel’ of clothes. Nightwear and pyjamas are often seen as ‘comfy’ clothes, as this care‐worker reflects. However, pyjamas or dressing gowns were generally discouraged and seen as inappropriate for public areas of the home, as it ‘looks like we haven't bothered’. One resident, Rosemary, described how she used to feel ‘comfortable’ wearing her dressing gown in the evening at home when she came in from work, but felt care‐workers discouraged this and ‘like you to wash and dress’.

An emphasis on visual appearance can therefore undermine care as an ‘art of dwelling’ enacting a sense of ‘being‐at‐home’ through engagements with familiar things (Schillmeier and Domènech 2009: 288). Old and tatty clothes were discouraged as they looked ‘uncared’ for. But sometimes older ‘worn’ clothes were those people with dementia felt most at ease in. Often participants had a favourite item of clothing which was worn and re‐worn. As one woman with dementia said: ‘I think it's what you get used to and what you like …. what you feel comfortable in.’ Woodward (2007: 78) notes that clothing which is repeatedly worn becomes more ‘comfortable’ as it is softened by the body, and becomes a ‘second skin’, part of the wearer.

Tensions between logics of care and logics of choice (Mol 2008) were taken to the extreme where the person with dementia refused to change soiled clothing:

… you can't leave them because the family could turn up and then they're going to say, ‘Why is mum …’ you know, ‘covered in faeces?’ or, ‘all wet in her chair? … It is always complicated because you don't know what … Because it's against their will, they don't want to do it but on the other hand we're not supposed … its neglect. It's abuse. You can't just leave them dirty or wet. [Michael, care‐worker]

This was described as a ‘catch 22 situation’‐ if you leave the person in soiled clothing it is ‘neglect’ and detrimental to their health, if you force them to change then it is abuse. These situations required careful practices of emotion work, as discussed above. Care‐workers sometimes engaged in ‘tinkering’ (Mol *et al*. 2010) to find individualised clothing solutions that met institutional aesthetic standards (Pols 2013). For instance, buying multiple ‘orange cardigans’ for a woman who wanted to ‘wear the same cardigan all the time’ so she could still be ‘clean and tidy’. Care home manager Anita argued that such decisions need a ‘very individualised sort of assessment’ in order to keep the person ‘comfortable and okay with their world.’ This involved taking into account biographical knowledge of the person, and interpreting their wishes through observing their ‘behaviour and moods’ where verbal communication is impaired. In some cases this means making room for non‐normative bodies, for instance, care‐workers and relatives accepted Bobby being ‘scruffy’ because he has ‘always been scruffy’. On the other hand, another family carer was devastated to find her father looking ‘scruffy’, because it was viewed as a betrayal of who he was:

I've never seen my dad scruffy. Never. Until that day I turned up in the home and he's sitting there in screwed up clothes which really hurt me because I'm not used to that – not at all. [Melissa, family carer]

Therefore, standardised measures of care need to take into account ‘biographies and individualities’ in judging the aesthetics of care (Schillmeier 2014: 122), and make room for bodies and material practices which are outside of institutional aesthetic norms (Pols 2013). It is thus important to distinguish between ‘care‐less’ dress practice (Lavis *et al*. 2015: 2), and practice which creates room for different ways of being.

## Conclusion

We have argued for the importance of greater dialogue between literature in dress studies, and sociological research on health, illness and care. Drawing on the tradition in material culture studies of using detailed analysis of an artefact to explore wider cultural meanings, we have used dress as a route into exploring wider ambiguities and complexities relating to care in practice. The material properties of dress afforded different possibilities for how care was enacted, shaping experiences of daily routines, and creating potential for supporting identity through clothing that looks and ‘feels’ right. In turn, care shaped dress practice, with the physical challenges of dressing the body, and institutional laundry regimes constraining the possibilities.

An examination of the materiality of dress helps bring to light practices of care as tangible, concrete, and embodied, but also raises dilemmas and tensions regarding the visibility and aesthetics of care. It illuminates how care‐workers are caught between working with the body as the object of functional care, and the body as a materialization of personhood (Wolkowitz 2002). The time pressures of institutional routines, and pressure to maintain presentable dress can lead to bodies becoming objectified and standardised. Furthermore, a focus on the production of bodies and maintaining physical health which still remains at the heart of the day to day practice of care means that clothing can fall down the list of priorities.

As a result, we propose a ‘reimagining’ of care, locating clothing in a broader movement to shift the emphasis away from functional care, towards its relational and processual elements (Latimer 2013). This approach requires a rethinking of bodies and bodywork, challenging the focus on the physical body as the object of care, and emphasising the body as a materialisation of personhood. Rather than an activity to be rushed, the act of dressing can be an opportunity for ‘being with’, a time for one‐to‐one interaction, sensory engagement, and a practice of supporting identity. As an aspect of bodywork, dressing also involves emotion work, not only in terms of the use of emotional labour to ‘get the job done’ (Lee‐Treweek 1996), but in some cases also genuine emotional attachments, with dress entangled with a sense of ‘comfort’ and ‘caring about’ the person.

Making dress a priority is a challenge which requires change not only at the level of individual care homes and day‐to‐day practice, but also at the level of policy. At present there is a lack of detailed guidance and training for care‐workers on dress or appearance. Although National Care Standards for nursing homes briefly mention that residents should always be dressed in ‘their own clothes’ and clothing of ‘their choice’ where possible (Department of Health 2015: 28), there is no guidance on practices of dressing, or how to support choice. Managers felt there was a greater emphasis in CQC inspections on maintaining a dignified and presentable appearance and on issues related to hygiene, rather than individualised and personalised dress. While such regulation is important for challenging the neglectful appearance associated with earlier institutional dress (Twigg 2010), it can have the effect of reducing dress to a tick box, a visible indicator of care. Further guidance and training for care‐workers which recognises the significance of appearance in terms of identity, biography and sensory engagement would help address this. As we have argued along with colleagues (Campbell *et al*. 2015), tools such as storyboards in residents’ rooms could help to provide information about appearance biographies and preferences in a more accessible way, facilitating the ‘curation of identity’ (Crichton and Koch 2007). Such approaches could help challenge standardisation of dressed bodies, contributing to more sensitive and personalised care practice.
